# Socioeconomic disparities in gait speed and associated characteristics in early old age

**DOI:** 10.1186/s12891-016-1033-8

**Published:** 2016-04-23

**Authors:** S. Plouvier, M. Carton, D. Cyr, S. Sabia, A. Leclerc, M. Zins, A. Descatha

**Affiliations:** Inserm UVSQ, Population-based Epidemiologic Cohorts Unit, UMS 011, F-94807 Villejuif, France; Versailles St-Quentin University, UMS 011, UMR-S 1168, F-94807 Villejuif, France; Inserm UVSQ AP-HP, VIMA: Aging and chronic diseases. Epidemiological and Public Health Approaches, U1168, occupational health Unit, Poincaré University Hospital, F92380 Garches, France; Department of Epidemiology & Public Health, University College London, London, UK; Versailles St-Quentin University, Boulogne-Billancourt, France

**Keywords:** Usual gait speed, Socioeconomic position, Occupational exposure

## Abstract

**Background:**

A few studies have documented associations between socioeconomic position and gait speed, but the knowledge about factors from various domains (personal factors, lifestyle, occupation…) which contribute to these disparities is limited. Our objective was to assess socioeconomic disparities in usual gait speed in a general population in early old age in France, and to identify potential contributors to the observed disparities, including occupational factors.

**Methods:**

The study population comprised 397 men and 339 women, aged 55 to 69, recruited throughout France for the field pilot of the CONSTANCES cohort. Gait speed was measured in meters/second. Socioeconomic position was based on self-reported occupational class. Information on personal characteristics, lifestyle, comorbidities and past or current occupational physical exposure came either from the health examination, from interview or from self-administered questionnaire. Four groups were considered according to sex-specific distributions of speed (the two slowest thirds versus the fastest third, for each gender). Logistic regression models adjusted for health screening center and age allowed to the study of cross-sectional associations between: 1- slower speed and occupational class; 2- slower speed and each potential contributor; 3- occupational class and selected potential contributors. The association between speed and occupational class was then further adjusted for the factors significantly associated both with speed and occupational class, in order to assess the potential contribution of these factors to disparities.

**Results:**

With reference to managers/executives, gait speed was reduced in less skilled categories among men (OR 1.21 [0.72–2.05] for Intermediate/Tradesmen, 1.95 [0.80–4.76] for Clerks, Sale/service workers, 2.09 [1.14–3.82] for Blue collar/Craftsmen) and among women (OR 1.12 [0.55–2.28] for Intermediate/Tradesmen, 2.33 [1.09–4.97] for Clerks, 2.48 [1.18–5.24] for Sale/service workers/Blue collar/Craftsmen). Among men, occupational exposure to carrying heavy loads explained a large part of socioeconomic disparities. Among women, obesity and occupational exposure to repetitive work contributed independently to the disparities.

**Conclusions:**

This study suggests that some potentially modifiable occupational and personal factors explain at least part of the differences in gait speed between occupational classes, and that these factors differ between men and women. Longitudinal studies are needed to confirm and complement these findings.

## Background

Various studies have focused on socioeconomic disparities in either self-reported or measured physical capability, such as walking speed, both for older adults and for those in early old age. Among studies dealing with walking speed or other objective measures of physical performance/strength, results differed according to the outcome of interest, the population considered, and the indicator of socioeconomic position: level of education, childhood socioeconomic position, wealth, or an indicator taking into account occupational category. In a large sample of Swedish men and women aged 60 or more, educational disparities were identified in lower body physical functioning (measured with chair stand test), walking speed and balance for both sexes, and in upper-limb strength (measured using grip strength test) for women only [1]. After adjustment for various chronic diseases, lifestyle and individual factors, significant disparities remained in walking speed for both sexes, in balance and grip strength for women and in chair stand for men. In the whole sample, differences in balance, chair stand and walking speed were significant only for those under age 80. In Italy, significant disparities in grip strength and performance to a physical battery score (including walking speed, balance, and lower physical functioning) were observed between manual and non-manual workers aged 80 or more after adjustment for demographic, education, cognition, lifestyle, sensorial and health factors [2]. Physical stress at work also appeared to play a role in the observed disparities. Among 53 year-old British, disparities in balance and lower physical functioning were observed between manual and non-manual workers, while no difference could be seen in grip strength [3]. After adjustment for physical activity, individual and health factors, disparities remained significant excepted for lower physical functioning for men. Among European subjects from the SHARE study, aged at least 50 years, education, occupational class, income and wealth were associated with upper-limb strength among men. For women associations were observed with education and wealth [4]. Health explained part of the associations, but the association with wealth remained significant after adjustment for health measures. In the English Longitudinal Study of Ageing, accounting for socio-demographic, behavioral and health factors, significant differences in walking speed remained between the richest and the poorest subjects [5]. Childhood socioeconomic position may also contribute to physical performance [6, 7]. In a meta-analysis, even after adjustment for adult socioeconomic position and body size (height and weight or body mass index (BMI)), an association of childhood socioeconomic position with walking speed and lower physical functioning was still present [6]. A gradient in upper limb strength, lower physical functioning and balance across childhood as well as adult socioeconomic position was also reported among UK subjects aged 60–64 years [7].

The potential role of specific occupational exposures in the past, in addition to lifestyle and health factors, remains unclear. In a large sample of Finnish men and women aged 55 years or older, educational disparities were observed in walking speed, climbing and balance for both sexes and also in squatting for women [8]. Detailed results for climbing indicated that the disparities were largely due to a higher prevalence of obesity (especially for women), smoking (for men), chronic conditions and physically strenuous work, among the less educated subjects. Among British civil servants aged 50 to 73 years, walking speed was related to employment grade. Physical functioning, financial insecurity and height appeared to be important explaining factors. However, more than half of the social gradient remained unexplained after considering various individual, lifestyle, psychosocial, health, employment and functional factors. The role of physical occupational factors was not studied, since most British civil servants were not exposed to them [9].

Among measures of physical capacity, walking speed has been considered as an important component of frailty in older adults [10]. Gait speed at usual pace, a tool for assessment of vulnerability to adverse outcomes in older adults, is also considered as an important dimension of physical capability in early old age [8, 9, 11, 12]. The objective of the present study was to assess the associations between usual gait speed and occupational class in early old age in a general population and to explore the personal and occupational factors which could play a role as confounding or intermediate factors in this association.

## Methods

### Population

#### The CONSTANCES cohort

The CONSTANCES cohort project aims at providing a general prospective cohort of a large sample of the French population aged 18–69 years old [13, 14]. Participants are recruited among people affiliated to the main national health insurance provider, which covers more than 80 % of the French population. The cohort is designed to be representative of the target population according to age, sex, occupational status, and occupational class.

A random sample from the target population (see Constances.fr) is invited by mail to join the cohort. Those who agree have to fill two self-administrated questionnaires dealing with lifestyle, health, physical limitations, social and personal characteristics, and lifetime job history. They are invited to go to one of the nineteen participating Health Screening Centers (HSC) throughout France, in order to benefit from an extensive health examination (medical, paraclinic exams, blood tests). In addition, the participant’s personal and familial clinical history is recorded by a physician, and additional tests on physical and cognitive status are performed for those aged 45 years or more. Specific themes are also explored: detailed information on past and current occupational exposures is collected by trained investigators.

Follow-up will include annual self-administered questionnaires and, every five years, a visit to the HSC. Additional data on social and work-related events and on health will be collected from national databases which contain information on occupational status and retirement, health and mortality. Inclusion began in 2012 and 200 000 participants are expected to be included. Beside its general purpose, this cohort will focus on occupational and social determinants of health, ageing and women’s health. The results presented here are based on the first available data, those from the field pilot.

### The field pilot

Before inception, a field pilot was performed in 2009–2010 in seven HSC throughout France, based on a specific random sample [13, 15]. Two self-administered questionnaires were mailed to the volunteers. At the HSC, a health examination was performed and, for those aged 55 years or more, physical and cognitive tests were administered. Specific themes were also explored.

This pilot study included approximately 3500 subjects, close to the general population of adults in France as to sex, age and socioeconomic status [13]. Among them, 843 were aged at least 55 years. The first descriptive analyses of this sample showed a diverse distribution of occupations, working conditions and lifestyle. Prevalence of various diseases and symptoms in this sample was close to that observed in other national surveys in France.

Only slight changes in the study design occurred between the pilot and the next steps of the project.

### The study population

The study population comprised 397 men and 339 women participating in the field pilot, aged 55 to 69 years when the self-administrated questionnaires were sent, whose occupational class was documented, and who had performed the gait speed test.

### Data

#### Usual gait speed

Time needed to walk three meters with comfortable shoes was measured with a stopwatch. One meter zones were added on either side in order to accelerate and to decelerate before and after the start and stop lines. Subjects were asked to walk at their normal, usual speed. One trial was allowed before measurement. Speed was expressed in meter per second. Two groups were considered in each gender: those whose gait speed was in the upper tertile of the sex-specific distribution of speed - reference - versus the others, referred to as “slower speed” in the following text. Comparing the upper tertile to the others was preferred to considering gait speed as a quantitative variable, because we were not interested in the speed itself, but rather to compare to a reference situation, belonging to the upper tertile of the distribution.

### Occupational class

Self-reported information collected from the lifestyle and job history questionnaires was used to classify subjects according to the French national classification of jobs and socioeconomic categories [16]. The current Occupational Class (OC) was retained for those still working and the longest held one for retirees. Men were classified in four categories: 1- Manager or executive; 2 - Intermediate or tradesman; 3- Clerk, sales or service worker; 4- Blue-collar worker or craftsman. Categories for women were slightly different, due to the large proportion of clerks and the relatively small proportion of blue-collar workers among them: 1- Manager or executive; 2- Intermediate or tradesman; 3- Clerk; 4- Sales or service worker, blue-collar worker or craftsman. The categories used for men and women correspond to the usual classification in France. Men and women in category 1 have the better average level of education, followed by those in category 2. Subjects in categories 3 and 4 are less skilled and have a lower level of education.

Two factors were considered as covariates in the analyses:

- age (when gait speed was measured), in two classes: sixty years old or less versus older than 60;
- HSC in seven categories. An adjustment for Health Screening Center was preferred in order to avoid potential effects of physical and organizational conditions of the measure.

The following potential confounding factors for the association between OC and gait speed were considered

- Personal factors
  - Self-reported sports activities: never, two hours weekly or less, more than two hours weekly;
  - Height, dichotomized at the median: smaller than 1.73 m versus 1.73 m or more for men; and 1.60 m or smaller versus more than 1.60 m for women;
  - Self-reported smoking status: never smoked versus ever;
  - Body mass index (BMI), calculated as weight (kg) divided by height (m^2^) with height and weight measured by a trained nurse was categorized as: < 25 Kg/m^2^, normal weight; 25 to 30 Kg/m^2^, overweight ; and ≥30 Kg/m^2^, obesity;
- Health related factors
  Presence or absence of cardiovascular disorders (at least one of the following: hypertension, arteritis, coronary heart disease, stroke) and of musculoskeletal disorders (at least one of the following: rheumatoid arthritis, osteoarthritis, sciatica, back pain, osteoporosis) reported at the health examination;
- Occupational factors
  Two occupational exposures ever encountered during the working life, assessed by interview: carrying heavy loads (ever/never) and repetitive work (ever/never). Repetitive work was defined as having to repeat the same action several times per minute.

### Analyses

Analyses were performed separately for men and women using logistic regression models for dichotomous outcomes and polytomous models for outcomes in more than two categories. Age and HSC were adjusted for in all analyses.

The first analyses focused on the association between gait speed (“slower” versus upper tertile) and OC, in order to document occupational disparities in gait speed (first model).

In a second step, associations between gait speed and each individual or occupational characteristic were studied, in order to identify factors which might play a role in the association observed in the previous step. The variables globally associated with gait speed at a *p*-level < 0.05 and those for which one response category was associated with gait speed at a *p*-level < 0.05 were selected for the next steps.

Associations between these selected variables and OC were then assessed (results given in appendix). Those associated with OC (globally, or for a specific response category) at a *p*-level < 0.05 were considered as potential explaining factors (confounding or intermediate factors), potentially contributing to the relation between gait speed and OC.

These variables were then added to the first model: first only those classified as personal characteristics; then only occupational characteristics; and a final model with all the variables (personal and occupational) which could explain the associations observed in the first model.

In addition, we checked that the results were unchanged if some variables were added in the final model, those which were risk factors for a slower gait speed, but not significantly associated with OC.

## Results

### Population

Among 441 eligible men, 397 (90.0 %) were included in the study (20 did not undergo any of the testing for seniors; 20 did not take the gait speed test, and OC was missing for 4 men). Among 402 women, 339 (84.3 %) were included (20 did not undergo any of the testing for seniors; 13 did not take the gait speed test, and OC was missing for 30 women).

The median usual gait speed was 1.17 m/s (mean 1.20, SD 0.23) among men and 1.11 m/s (mean 1.12, SD 0.24) among women. Among men, 129 were in the upper tertile of the distribution of speed (speed > 1.25 m/s). The corresponding number for women was 122 (speed > =1.20 m/s).

The most common OC among men was managers/executives. Few men were clerks, sales or service workers (Table 1). Those in this class were mainly clerks or sales workers. The most common OCs among women were intermediate, mainly nurses, and the fourth class, which included mainly childcare workers, cleaners, sales workers, nurses aids, and blue-collar workers. Individual and occupational characteristics of the study population are presented in Table 1.

**Table 1:** Description of the population

		Men		Women	
		*N*	%	*N*	%
Usual gait speed	Two lower tertiles (slower speed)	268	67.5	217	64.0
	Upper tertile	129	32.5	122	36.0
Occupational class	Manager/executive	157	39.6		
	Intermediate grade/tradesmen	117	29.5		
	Clerks, sales/service workers	33	8.3		
	Blue collar/Craftsmen	90	22.7		
	Manager/executive			53	15.6
	Intermediate grade/tradesmen			101	29.8
	Clerks			86	25.4
	Sales/service workers/Blue collar/Craftsman			99	29.2
Age	55–60	180	45.3	174	51.3
	61–69	217	54.7	165	48.7
Sport	Never	129	32.5	112	33.0
	<=2 hours/week	115	29.0	123	36.3
	>2 hours/week	153	38.5	104	30.7
BMI*	Normal weight	145	36.5	183	54.0
	Overweight	192	48.4	113	33.3
	Obese	60	15.1	43	12.7
Height	Average**	192	48.4	183	54.0
	Taller***	205	51.6	156	46.0
Smoking	Ever	232	58.4	109	32.2
	Never	165	41.6	230	67.8
Cardiovascular disorders	Absence	293	73.8	261	77.0
	Presence	104	26.2	78	23.0
Musculoskelatal disorders	Absence	332	83.6	246	72.6
	Presence	65	16.4	93	27.4
Carrying heavy loads	Ever exposed	129	32.5	107	31.6
	Never exposed	268	67.5	232	68.4
Repetitive work	Ever exposed	48	12.1	50	14.7
	Never exposed	349	87.9	289	85.3

*Standard=less than 25 Kg/m^2^; overweight=from 25 to less than 30 Kg/m^2^; obese=at least 30 Kg/m^2^

**Less than 1.73 for men; 1.60 m or less for women

***1.73m or more for men; more than 1.60m for women

### Occupational class, gait speed and personal and occupational characteristics

#### Men

Controlling for age and HSC, a slower gait speed was significantly more frequent for blue collar workers/craftsmen than for managers/executives, with category 2 (Intermediate grade/tradesmen) in an intermediate situation (Table 2).

**Table 2:** Associations between a slower usual gait speed and occupational class, adjusted for age and Health Screening Center

	Slower usual gait speed							
	Men				Women			
	OR	95 % CI		*p*	OR	95 % CI		*p*
Occupational class				0.077				0.012
Manager/executive	1				1			
Intermediate grade/tradesmen	1.21	0.72	2.05		1.12	0.55	2.28	
Clerks, sales/service workers	1.95	0.80	4.76					
Blue collar/Craftsmen	2.09	1.14	3.82					
Clerks					2.33	1.09	4.97	
Sales/service workers/Blue collar/Craftsmen					2.48	1.18	5.24	

A slower gait speed was more frequent among obese men, those who suffered from cardiovascular disorders, from musculoskeletal disorders and those who ever carried heavy loads (Table 3, all *P* < 0.05).

**Table 3:** Associations between a slower usual gait speed and individual and occupational factors considered separately, adjusted for age and Health Screening Center

		Men				Women			
		OR	95 % CI		*p*	OR	95 % CI		*p*
Sport	Never	1			0.323	1			0.130
	<=2 h/week	0.75	0.43	1.32		0.73	0.41	1.29	
	>2 h/week	0.67	0.40	1.14		0.54	0.30	0.98	
BMI^a^	Normal weight	1			0.059	1			0.026
	Overweight	1.42	0.88	2.30		0.98	0.58	1.64	
	Obese	2.34	1.14	4.81		3.31	1.35	8.13	
Height	Standard^b^	1			0.900	1			0.123
	Taller^c^	0.97	0.63	1.51		0.69	0.43	1.11	
Smoking	Never	1			0.222	1			0.319
	Ever	1.32	0.85	2.05		0.77	0.47	1.28	
Cardiovascular disorder	Absence	1			0.007	1			0.608
	presence	2.09	1.22	3.58		0.87	0.50	1.51	
Musculoskeletal disorder	Absence	1			0.042	1			0.388
	Presence	1.93	1.02	3.65		1.27	0.74	2.20	
Carrying heavy loads	Never exposed	1			0.015	1			0.076
	Ever exposed	1.84	1.13	3.01		1.61	0.95	2.71	
Repetitive work	Never exposed	1			0.408	1			0.049
	Ever exposed	0.76	0.40	1.46		2.12	1.00	4.46	

^a^Standard = less than 25 Kg/m^2^; overweight = from 25 to less than 30 Kg/m^2^; obese = at least 30 Kg/m^2^

^b^Smaller than 1.73 for men; 1.60 m or smaller for women

^c^1.73 m or more for men; more than 1.60 for women

Among these three variables associated with a slower gait speed, only “ever carried heavy loads” was also associated with OC; this occupational exposure was less frequent among managers or executives (Appendix).

When this occupational strain was added to the first model, the OR for blue collar/craftsmen was reduced to 1.65 [0.85, 3.23] (Tables 4 and 5). The main results were unchanged when BMI or health variables were added to the final model.

**Table 4:** Associations between a slower usual gait speed and occupational class, individual and occupational factors, adjusted for age and Health Screening Center: Men

		OR	95 % CI		*p*
Occupational class					0.354
	Manager/executive	1			
	Intermediate/tradesmen	1.12	0.66	1.92	
	Clerks, sales/service workers	1.81	0.74	4.44	
	Blue collar/Craftsmen	1.65	0.85	3.23	
Carrying heavy loads	Never exposed	1			0.124
	Ever exposed	1.54	0.89	2.65

**Table 5:** Associations between a slower usual gait speed and occupational class, individual and occupational factors, adjusted for age and Health Screening Center: Women

		Model 1				Model 2				Model 3			
		OR	95 % CI		*p*	OR	95 % CI		*p*	OR	95 % CI		*p*
Occupational class					0.038				0.043				0.087
	Manager/executive	1				1				1			
	Intermediate/tradesmen	1.07	0.52	2.18		1.10	0.54	2.25		1.05	0.51	2.16	
	Clerks	2.21	1.03	4.75		2.27	1.06	4.84		2.17	1.01	4.66	
	Sales/service workers/blue collar/Craftsmen	2.09	0.98	4.47		2.12	0.97	4.66		1.79	0.80	4.00	
BMI					0.070								0.073
	Normal	1								1			
	Overweight^a^	0.93	0.55	1.58						0.91	0.54	1.54	
	Obese^b^	2.79	1.11	7.02						2.74	1.08	6.94	
Repetitive work	Never exposed					1			0.2466	1			0.266
	Ever exposed					1.61	0.72	3.61		1.60	0.70	3,66	

^a^from 25 to less than 30 Kg/m^2^; ^b^at least 30 Kg/m^2^

#### Women

Controlling for age and HSC, a slower gait speed was significantly associated with OC. It was significantly more frequent in the less skilled occupational categories (Table 2).

A slower usual gait speed was also less frequent in the third category for sport (> two hours of sports per week), and more frequent for obese subjects. An association with repetitive work was also found (Table 3). Among those variables associated with a slower gait speed, BMI and exposure to repetitive work were also associated with OC: women in the less skilled category were more frequently obese, and more frequently ever exposed to repetitive work (Appendix).

When BMI only was added to the first model (Tables 4 and 5), occupational disparities for gait speed remained, with an elevated OR for clerks and those in the less skilled category. When only ‘ever had a repetitive work’ was added to the first model, the association with OC also remained significant.

When both BMI and ‘ever had a repetitive work’ were added to the first model, the link with OC was less strong (*p* = 0.087). Being a clerk remained a risk factor for slower usual gait speed. In this model, obesity remained also a risk factor.

Adding sport (in 2 classes) to the final model had consequences for the role of obesity (which was no longer significant), but the association with OC tended to be slightly stronger.

## Discussion

Occupational disparities in usual gait speed in early old age, to the detriment of the lower occupational classes were identified in the general population in France. These disparities were partly reduced when potentially modifiable personal and/or occupational factors acting as confounding (or intermediate) factors were considered, with some differences between genders.

### Methodology

Some variables in this study were measured variables, such as height and weight. Health information was documented by the examining physician using both open and closed questions. Other variables were self-reported. For OC, consistency between information issued from the lifestyle and the job history questionnaires was checked. In this study the lifetime working history (self-reported) was considered in order to include retired subjects, and to minimize selection bias, since those still exposed in early old age could be healthier (healthy worker effect). OC could be studied in a general population in a rather precise and reliable way, with four different strata. In addition, participation rate was rather good and bias selection was likely to be reduced as usual gait speed for people without information on OC appeared similar to that of people with known OC.

Usual gait speed was measured according to a standardized protocol by trained personnel. Observed values could appear lower than those observed in other studies, or proposed as references for ‘healthy’ subjects [9, 17, 18]. Comparisons between studies are difficult, due to differences in methods (distance, acceleration allowed or stop and go, stopwatch or photocell…). Populations could also differ. However, despite difficulties in comparing results, measure of usual gait speed is generally considered as reliable and easy to perform [11, 17]. The reliability and validity of the measure has most often been assessed over 4 meters [11]. Here, gait speed was measured over three meters. This is less usual, but distances shorter than 4 meters are also used: in another study, the time needed for walking 8 feet (2.44 meters) was a good predictor of incident disability [19].

The first objective of this study was to assess whether gait speed was associated with OC. The second was to explore the role of factors which could “explain” an observed association: those factors may be confounding factors (independent risk factors for gait speed, also associated with OC), or intermediate factors (consequence of OC, proximal factors for gait speed). Occupational factors could be considered more as intermediate factors, and personal factors as independent confounding factors. But discriminating between confounding and intermediate factors is partly arbitrary.

### Findings

Having a slower gait speed was associated with OC, to the detriment of the lower occupational classes, which is consistent with other studies [1, 5, 9]. In this study, as expected, women walked slower than men and older people, quite slower than younger people [17, 18]. Physical exercise in general is known to improve performance. Those who declared doing sports rather often tended to be faster walkers, especially women. Such an association has already been reported [1, 9]. In a meta-analysis, therapeutic exercise was shown to have a significant effect on usual gait speed among people aged 60 years or more [20]. A modest improvement in gait speed among older people after progressive resistance strength training has also been reported [21]. Nonetheless, those walking faster could also be those more interested in engaging in sports activities. The association observed between usual gait speed and obesity has been observed before, mainly in cross-sectional studies [22]. In addition to obesity, other aspects could be important, such as muscle strength, body composition or waist circumference [23–27]. In contrast to previous reports [9, 18, 28], height was not associated with gait speed in our study, especially among men, which could be due to a small range for height. Gait speed has been related to various chronic conditions [1, 29]. However in our study an association with health variables was observed only among men. To our knowledge this is the first study investigating the association between gait speed and specific occupational physical exposure.

Among the sex-specific factors significantly associated with a slower usual gait speed, few were associated with OC. Sport was not, at least among women. Nonetheless, a higher level of leisure-time physical activity in higher socio-economic categories at working age was already reported [30]. In France, among people aged 45 to 68, meeting the recommended level of physical activity is more common among those aged at least 60, and among women with a higher level of education [31]. BMI was associated with OC among women, but not among men. This is consistent with the situation in France and in other developed countries [32]. Socioeconomic inequalities in cardiovascular diseases and musculoskeletal disorders have been described [33–35]. However, they were not observed among men in this study, except for a higher frequency of cardiovascular disorders among blue collar workers. Finally, associations between OC and occupational exposures were consistent with what would be expected.

The study suggests that some, still frequent, occupational exposures or personal factors might explain at least part of the difference in gait speed between occupational classes. These factors differ between men and women, which is not very surprising, especially for occupational factors.

Occupational exposure (more precisely, carrying heavy loads) seems to play a major role in disparities in gait speed across occupational classes among men. This could appear as surprising, but a negative role of past exposure to manual work has also been found for physical function in people aged 80 or older [2]. This is consistent with the fact that, whereas leisure-time physical activity has positive effects on health, occupational physical activity has detrimental effects [36]. Among women, both occupational exposure (repetitive work) and obesity appeared to play a role in gait speed disparities.

The results of this study can be compared only partly with those from other studies, due to differences in the list of explanatory factors. The role of BMI is consistent with observations in other studies or in the general population, concerning social inequalities in obesity. Our results on the role of chronic diseases do not differ much from the modest role of chronic diseases observed both in the Whitehall study and in a study in Sweden [1, 9]. However, the role of chronic diseases, especially cardiovascular diseases, musculoskeletal disorders and respiratory diseases, remains an hypothesis to be tested on large samples with a precise assessment of these specific health dimensions.

In this study, two limitations are the relatively small sample size, especially in some categories, and the cross-sectional design which implies cautious interpretation concerning causal inference. Domains not considered need also to be explored, especially those related to the results for clerks. Domestic activities have not been taken into account here, whereas they may play a role. Sedentarity at work could also affect gait speed, which would be consistent with the results found among clerks.

## Conclusion

This study suggests that some potentially modifiable occupational and personal factors explain at least part of the differences in gait speed between occupational classes, and that these factors differ between men and women. These results are in accordance with those from some other studies, which indicate that work-related physical activity has negative consequences for physical capability in early old age. This has consequences in terms of prevention at the workplace, and for public health recommendations such as the importance of leisure-time physical activity. Longitudinal studies based on larger populations are needed for a better understanding of the factors important for gait speed, and more generally for physical capability.

### Ethics and consent to participate

Authorization from the appropriate ethics committee was obtained (« Commission nationale de l'informatique et des libertés » n° 910486, “Comité consultatif sur le traitement de l’information en matière de recherche » 10.628). The study is in compliance with the Helsinki Declaration, and informed consent for participation in the study was obtained from participants.

## Availability of data and materials

All Constances data available on request to Dr M. ZINS, head of the Inserm UMS 011 (http://www.constances.fr/index_EN.php).

## Appendix

**Table 6:** Occupational class and selected individual and occupational factors (1), adjusted for age and Health Screening Center

	Occupational class				
MEN	Manager/executive	Intermediate grade/tradesmen	Clerks, sales/service workers	Blue collar/Crafstmen	
Outcomes	ref	OR 95 % CI	OR 95 % CI	OR 95 % CI	P
Overweight^a^	1	1.03 0.60 1.75	0.79 0.35 1.78	1.24 0.68 2.26	0.743
Obese^b^	1	1.10 0.51 2.37	0.53 0.14 2.05	1.71 0.77 3.79	
Cardiovascular disorders	1	1.06 0.60 1.86	0.68 0.26 1.79	1.62 0.91 2.90	0.239
Musculoskeletal disorders	1	0.61 0.31 1.23	0.30 0.07 1.35	1.09 0.56 2.11	0.193
Ever carried heavy loads	1	3.13 1.68 5.82	2.97 1.22 7.25	15.1 7.83 29.2	<0.0001
WOMEN	Manager/executive	Intermediate grade/tradesmen	Clerks	Sales/service workers/Blue collar/Craftsmen	
Outcomes	ref	OR 95 % CI	OR 95 % CI	OR 95 % CI	P
Sports < =2 h/week^c^	1	1.64 0.72 3.76	1.19 0.52 2.73	0.79 0.35 1.78	0.461
Sports >2 h/week^c^	1	1.61 0.69 3.78	0.94 0.39 2.27	0.84 0.36 1.94	
Overweight^a^	1	0.77 0.37 1.59	1.12 0.53 2.35	1.04 0.49 2.21	0.002
Obese^b^	1	1.88 0.36 9.71	3.28 0.65 16.6	10.0 2.15 46.8	
Ever had a repetitive work	1	3.31 0.38 28.6	4.83 0.57 41.0	35.6 4.60 274	<0.0001

(1) Individual and occupational factors associated with usual gait speed, different for men and women.

^a^from 25 to less than 30 Kg/m^2^, compared to normal weight; ^b^at least 30 Kg/m^2^, compared to normal weight. ^c^compared to “never”.

## Abbreviations

BMI: body mass index
HSC: Health Screening Centers
OC: occupational class
OR: odds ratio
